# Genomic evidence of Y chromosome microchimerism in the endometrium during endometriosis and in cases of infertility

**DOI:** 10.1186/s12958-019-0465-z

**Published:** 2019-02-13

**Authors:** Muzaffer A. Bhat, Jai B. Sharma, Kallol K. Roy, Jayasree Sengupta, Debabrata Ghosh

**Affiliations:** 1 Department of Physiology, All India Institute of Medical Sciences, New Delhi, India; 2 Department of Obstetrics and Gynaecology, All India Institute of Medical Sciences, New Delhi, India

**Keywords:** Endometriosis, Endometrium, Infertility, Microchimerism, Y chromosome

## Abstract

**Background:**

Previous studies, which were primarily based on the fluorescent in-situ hybridisation (FISH) technique, revealed conflicting evidence regarding male foetal microchimerism in endometriosis. FISH is a relatively less sensitive technique, as it is performed on a small portion of the sample. Additionally, the probes used in the previous studies specifically detected centromeric and telomeric regions of Y chromosome, which are gene-sparse heterochromatised regions. In the present study, a panel of molecular biology tools such as qPCR, expression microarray, RNA-seq and qRT-PCR were employed to examine the Y chromosome microchimerism in the endometrium using secretory phase samples from fertile and infertile patients with severe (stage IV) ovarian endometriosis (OE) and without endometriosis.

**Methods:**

Microarray expression analysis followed by validation using RNA-seq and qRT-PCR experiments at the RNA levels and further validation at the DNA level by qPCR of target inserts for selected targets in eutopic endometrium samples obtained from control (CON) and stage IV ovarian endometriosis (OE), either from fertile (FCON and FOE; *n* = 30/each) or infertile (ICON and IOE; n = 30/each) women, were performed.

**Results:**

Six coding (AMELY, PCDH11, SRY, TGIF2LY, TSPY3, and USP9Y) and 10 non-coding (TTTY2, TTTY4C, TTTY5, TTTYY6, TTTY8, TTTY10, TTTY14, TTTY21, TTTY22, and TTTY23) genes exhibited a bimodal pattern of expression characterised by low expression in samples from fertile patients and high expression in samples from infertile patients. Seven coding MSY-linked genes (BAGE, CD24, EIF1AY, NLGN4Y, PRKY, VCY and ZFY) exhibited differential regulation in microarray analysis, and this change was validated by RNA-seq or qRT-PCR. DNA inserts for 7 genes in various samples were validated by qPCR. The prevalence and concentration of PCR-positive target inserts for BAGE, PRKY, TTTY9A and ZFY displayed higher values in the fertile, control (FCON) patients compared with the fertile, endometriosis patients (FOE).

**Conclusion:**

Several coding and non-coding MSY-linked genes displayed microchimerism as evidenced by the presence of their respective DNA inserts, along with their differential transcript expression, in the endometrium during endometriosis and in cases of infertility.

**Electronic supplementary material:**

The online version of this article (10.1186/s12958-019-0465-z) contains supplementary material, which is available to authorized users.

## Background

**Fig. 1:**
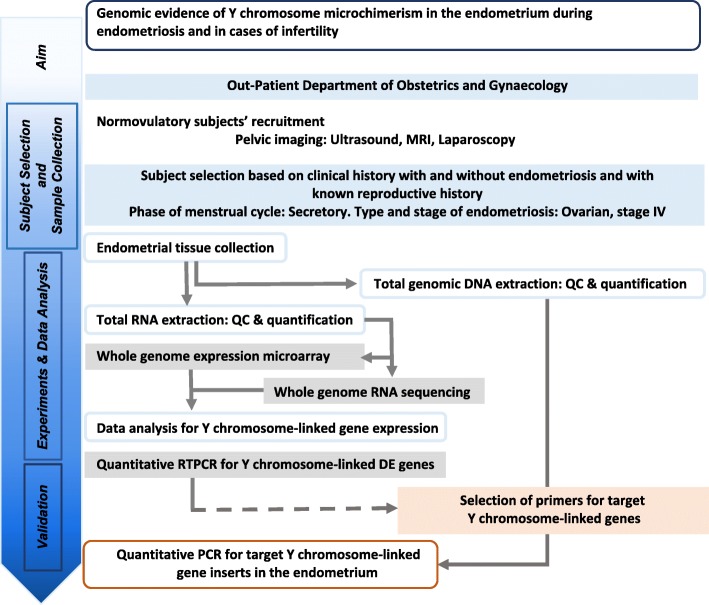
A schematic flow chart of the study design. Genomic evidence of Y chromosome microchimerism in the endometrium in endometriosis and infertility was probed first by examining Y chromosome-centric transcript expression data from whole genome microarray experiments and from whole genome RNA-seq data followed by validation at the RNA level by quantitative RT-PCR and the DNA level by quantitative PCR

Amongst several theories on the pathogenesis of endometriosis, Sampson’s theory postulating that viable endometrial cells deposited at ectopic sites due to retrograde menstruation implant, grow and eventually result in the histogenesis of endometriosis has received wide acceptance [1]. Although retrograde menstruation and exfoliated endometrial cells in the peritoneal cavity are reportedly present in most cycling women, approximately 10% of women in the reproductive age group suffer from endometriosis [1, 2]. It is therefore conjectured that there are other factors possibly at play in the endometrium that predispose a subgroup of women towards the histogenesis of endometriosis at ectopic sites. Several lines of evidence indeed support the notion that some intrinsic defects in the eutopic endometrium are responsible for the resultant endometriosis [3–5]. Substantial evidence suggest that women with endometriosis bear an increased risk of developing autoimmune diseases [6–8]. However, several autoimmune diseases are reportedly associated with male microchimerism [9–11]. Taken together, it appears that male microchimerism in the eutopic endometrium may be a supplementary factor towards the histogenesis of endometriosis at ectopic sites. A few transcripts related to Y-chromosomal genes were recorded in the secretory phase of eutopic endometrial samples obtained from patients with stage-confirmed endometriosis [12, 13]. Additionally, direct evidence of male microchimerism in endometriosis was earlier reported in a study [14]. Fassbender et al., however, failed to obtain evidence of such male microchimerism in the endometrium based on FISH-based experiments in a total of 31 patients, of which 19 had stage-confirmed endometriosis [15]. In the present study, we have examined this issue of Y-chromosomal microchimerism in fertile and infertile women with ovarian endometriosis (OE), without any reported uterine pathology. This was achieved using a sequence of experiments that included microarray expression followed by RNA-seq and quantitative RT-PCR experiments at the RNA levels and further validation at the DNA level by quantitative PCR of target inserts for selected targets (see Fig. 1 for the study design).

## Methods

### Patient selection and sample collection

Patients enrolled in the Department of Obstetrics and Gynaecology of the All India Institute of Medical Sciences New Delhi (AIIMS-D) for surgical intervention of endometriosis, in the Infertility Clinic or for family planning voluntarily took part in the study after understanding its purpose and giving written consent as per the standard protocol. The study was approved by the Institutional Ethics Committee on the Use of Human Subjects and conducted as per the Helsinki Declaration of 2013. There were two groups. The control group (CON; age: 35.7 ± 3.2 years) was comprised of proven fertile and normocyclic women who were undergoing either voluntary sterilisation, or hysterectomy for issues other than uterine diseases (FCON; *n* = 30), and women who were attending the infertility clinic (ICON; *n* = 30). The ovarian endometriosis group (OE; age: 37.2 ± 4.5 years) was comprised of women who were proven fertile (FOE; *n* = 30) or infertile, (IOE; *n* = 30) with confirmed stage IV ovarian endometriosis but having no report of recurrence of the disease or any other endocrinological disorder or other complications of comorbidities and who were enrolled for surgical intervention. Patients of both groups had normal BMI (20–25). Table 1 provides a summary of group-wise distribution of number of patients for different experiments, as well as the gender of offspring obtained from compliant women in FCON and FOE. The outline of the study design is shown in Fig. 1.

**Table 1:** Group-wise distribution of numbers of samples for different experiments

Group	Subgroup (*n*) [*m;n*]	Description	Samples used in experiments*			
			MA	RS	RT	PCR
Control	FCON (30)	Control, Fertile	9	5	5	6^a^
(CON)	[*12;3*]					
	ICON (30)	Control, Infertile	9	5	5	9^b^
Ovarian	FOE (30)	OE, Fertile	9	5	5	9^c^
Endometriosis	[*12;6*]					
(OE)	IOE (30)	OE, Infertile	9	5	5	9^c^

*Only quality assured samples were used. Additional archival samples: ^a^2, ^b^3, and ^c^4. MA, microarray experiment; FCON, control endometrium of fertile women; ICON, control endometrium of subfertile women; FOE, diseased endometrium of fertile women; IOE, diseased endometrium of sub-fertile women; RS, RNA-Seq; RT, qRT-PCR; PCR, quantitative polymerase chain reaction. *m*, number of fertile women reportedly having at least one male child; *n*, number of fertile women who declined to report details of children

### Tissue processing

Secretory phase endometrial samples were collected in PBS (pH 7.4) on ice and transported to the laboratory for immediate processing. Tissues to be used for qPCR, expression microarray, RNA-seq and qRT-PCR experiments were immediately processed for DNA and RNA extraction. RNA samples with a RIN score ≥ 8.0 were further used in expression microarray, RNA-seq and qRT-PCR experiments to determine the transcript levels as described below.

### Expression microarray and differential expression analysis

RNA extraction, followed by expression microarray experiments using 4 × 44 k whole genome expression microarray printed slides and Agilent platform and supplies (Agilent, Santa Clara, USA), was done as described in other studies [13, 16]. The high-resolution images were subjected to feature extraction using Agilent Feature Extraction software v10.7.3.1. Expression microarray data were log_2_ transformed and normalised to the 75th percentile for further analysis of expressed genes on the Y chromosome. The resulting gene lists were used as input data for statistical analysis as described below. The data have been archived at GSE120103.

### RNA-Seq

Nucleotide sequencing experiment for extracted RNA was done according to Trapnell et al. [17]. Total RNA samples were subjected to ribosomal RNA and other RNA removal using an oligo-dT for purification followed by fragmentation. Subsequently, they were copied into first strand cDNA using reverse transcriptase and random primers followed by a second strand cDNA synthesis using a reverse transcriptase enzyme. Fragments were purified with SPRI beads and subjected to an end-repair process. The cDNA libraries were generated following ligation with multiple indexing adapters to the ends of the ds-cDNA. Libraries were validated using BioAnalyzer (Agilent, Santa Clara, USA) after enrichment with PCR followed by pooling and normalisation. This was followed by enrichment of ligated cDNA molecules, preparing them for hybridisation onto a flow cell for the sequencing run on Illumina HiSeq2500 platform (Illumina, San Diego, CA, USA) using sequencing by synthesis chemistry. cDNA was passed through flow cells where it hybridised to the complementary sequences of adaptors. Amplification of the sequences was done using bridge amplification followed by clonal amplification and a sequencing reaction was performed using reversible dye termination chemistry following MINSEQE guidelines. The sequenced reads were obtained using the pair-end sequencing method and subjected to further analysis [17].

For analysis of RNA-seq data, the sequenced raw reads in FastQ format were first checked for low confidence bases, biased nucleotide composition, adapters, and duplicates and were further subjected to preprocessing. Q10 scores were determined from a Q table and poor-quality reads were filtered out from the downstream analysis using FastQC (Cambridge, CB22 3AT, UK). The selected reads were further subjected to alignment based on Human Reference Genome v19 using BowTie software (Baltimore, MD, USA), as described earlier [17, 18]. The referenced data in the BAM file was further analysed for its percentage match with the reference genome [19]. Only Y chromosome mapped reads were annotated and further selected, normalised and analysed as described below.

### Quantitative RT-PCR

Real time RT-PCR using SYBR-green chemistry was done using platform and chemicals obtained from BioRad (Hercules, CA, USA) to validate the differentially expressed transcripts obtained by expression microarray following the MIQE protocol [20]. Relative expression of selected genes along with that of endogenous control genes (B-ACTIN, B2M, GAPDH and UBC) in control and eutopic samples was assessed using BioRad CFX 96 Real Time PCR system. The methodological details have been described in other studies [13, 16, 21]. The primers for the target genes, as shown in Additional file 1 Table S1, were designed using Beacon Designer (Premier Biosoft, CA, USA) and obtained from Integrated DNA Technologies (Coralville, IA, USA).

### PCR detection for target DNA

DNA specimens extracted as previously described [22] were used for quantitative PCR to quantify the DNA copy numbers of the following genes: BAGE, EIF1AY, KDM5D, NLGN4Y, PRKY, TTTY9A, TTTY14, SRY and ZFY using SYBR-green chemistry with the abovementioned PCR system. Archival colon DNA (*n* = 4) and blood DNA (*n* = 4) samples from men were used as known positive control for Y chromosome genes. Quantitative assessment of DNA in terms of copy number of inserts were calculated using the standard curve constructed from the serially diluted PCR products of the ZFY gene segment of 490 bp at chromosomal location Yp11.2 [19]. The primers for the target genes, as shown in Additional file 1 Table S1, were designed using Beacon Designer software (Premier Biosoft, CA, USA). Specific nucleotide sequences were provided by Integrated DNA Technologies (Coralville, IA, USA).

### Data analysis

The microarray data were statistically analysed using Welch ANOVA followed by Tukey’s HSD test with Benjamini-Hochberg multiple testing corrections for false discovery rate to identify differentially expressed (DE) genes having > 2-fold at *P* < 0.05 in pair-wise comparisons. All data processing and analysis were performed using the GeneSpring software v14.9.1 (Agilent, Santa Clara, USA). Differential expression for RNA-seq data for Y chromosome mapped reads were analysed using the software DESeq v1.31.0 (Bioconductor Package, Buffalo, NY, USA). Differentially expressed genes were subjected to the Wald test followed by a Benjamini-Hochberg correction. A cut-off value of > 2-fold change and *P* < 0.05 were used to report the final list of significantly differentially expressed genes. Inter-group differences in quantitative RT-PCR were calculated as described previously [20]. For statistical analysis of prevalence, the Fisher’s exact test was performed. For non-Gaussian data, the Mann-Whitney U-test was applied, using SPSS v16.0 (IBM Analytics, New York, US).

## Results

### General

Table 1 reveals that approximately 40% of fertile patients with stage IV OE and with no uterine pathology reportedly had at least one male child, while 10% of fertile control patients and 20% of fertile patients with OE declined to provide the information about the gender of their children. However, as detailed in the following sections, endometrial samples from patients with and without endometriosis, irrespective of their clinical histories of pregnancy, displayed detectable DNA inserts and transcript expressions of Y chromosome-linked genes.

### Transcript expression

**Fig. 2:**
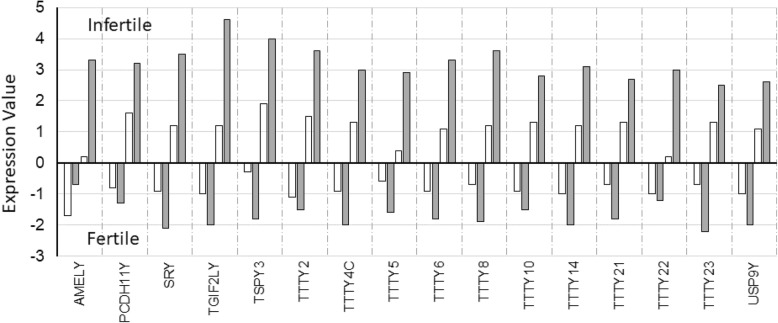
A typical bimodal pattern of transcript expression in data from microarray experiments characterised by low expression in samples from fertile patients and high expressions associated with infertility for six (6) coding (AMELY, PCDH11, SRY, TGIF2LY, TSPY3, and USP9Y) genes and ten (10) non-coding (TTTY2, TTTY4C, TTTY5, TTTYY6, TTTY8, TTTY10, TTTY14, TTTY21, TTTY22, and TTTY23) RNA genes. Control values are shown as *blank bars* and values for ovarian endometriosis are shown as *grey bars*

In whole genome expression microarray experiments, transcript expression of fifty-four (54) human male-specific regions of the Y chromosome (MSY) genes present in an ampliconic region of the Y chromosome were detected, and included 33 coding and 21 non-coding genes (Table 2). Interestingly, six (6) coding (AMELY, PCDH11, SRY, TGIF2LY, TSPY3, and USP9Y) and ten (10) non-coding (TTTY2, TTTY4C, TTTY5, TTTYY6, TTTY8, TTTY10, TTTY14, TTTY21, TTTY22, and TTTY23) genes exhibited a common bimodal pattern of expression characterised by low expression in samples from fertile patients while high expressions were associated with infertility (Fig. 2).

**Table 2:** Microarray expression levels of Y chromosome-linked genes in the endometrium^1^

Range of expression levels (in log2)	Descriptive scales	Groups (*n*)			
		FCON (9)	ICON (9)	FOE (9)	IOE (9)
−3.5 to − 2.0	Very low	0	0	15	2
<−2.0 to −0.5	Low	41	3	26	2
<−0.5 to 2.0	Moderate	11	49	13	4
> 2.0 to 3.5	High	2	2	0	34
> 3.5 to 5.0	Very high	0	0	0	12
Total^2^		54	54	54	54

FCON, control, fertile; ICON, control, infertile; FOE, stage IV, ovarian endometriosis, fertile; IOE, stage IV, ovarian endometriosis, infertile. ^1^data archived at GSE120103. ^2^Of 54 total genes showing RNA expression detected in microarray, 21 were for non-coding RNA (BCORP1, GOLGA2P2Y, **PRKY**, TTTY1, TTTY2, TTTY3, TTTY4C, TTTY5, TTTY6, TTTY7, TTTY8, **TTTY9A**, TTTY10, TTTY11, TTTY12, TTTY13, **TTTY14**, TTTY15, TTTY21, TTTY22 and TTTY23) and 33 were for coding RNA (AMELY, **BAGE**, BPY28, *CD24*, CDY2A, CSPG4P1Y, DDX3Y, DAZ2, **EIF1AY**, ERVH6, FAM19742, HSFY2, KDM5D, **NLGN4Y**, PCDH11Y, PRORY, PRY2, RBMY1B, RBMY 1E, RBMY 2EP, RPS4Y2, SRY, TBL1Y, TGIF2LY, TMSB4Y, TSPY3, TSPY4, TXLNGY, USP9Y, UTY, *VCY*, XKRY2 and **ZFY**). Genes shown in *bold* were confirmed by RNA-seq and qRT-PCR and those shown in *italics* were confirmed by qRT-PCR, but not by RNA-seq. For further details, see Additional file 2 Table S2

As Table 3 reveals, expression microarray experiments identified a total of 18 coding MSY-linked genes showing differential expression in different groups; 5 genes (BAGE, EIF1AY, NLGN4Y, PRKY and ZFY) of those 18 genes could be detected in both RNA-seq and real time RT-PCR, while expression of additional two (2) genes (CD24 and VCY) could be detected in real time RT-PCR, but not in RNA-seq. Thus, 11 coding genes (AMELY, BPY2B, CDY2A, DDX3Y, DAZ2, HSFY2, KDM5D, TBL1Y, TGIF2LY, TSPY3, and XKRY2) linked with the Y chromosome that exhibited a differential display in the between-groups comparisons of data obtained from expression microarrays, could not be detected by RNA-seq and quantitative RT-PCR. Twenty-one (21) non-coding Y chromosome-linked genes did not exhibit any differential display in the between-groups comparisons of data obtained from RNA-seq and RT-PCR (data not shown).

**Table 3:** Male specific Y chromosome (MSY)-linked coding genes showing differential expression (≥2-fold at *P* < 0.05) in microarray (MA), RNA-seq (RS) and qRT-PCR (RT) experiments

Comparison between groups	Gene symbol	Fold change		
		MA	RS	RT
FCON vs ICON	AMELY	−6.5	ND	ND
	CD24	−2.0	ND	NS
	CDY2A	− 6.1	ND	ND
	EIF1AY	−6.2	− 6.1	NS
	KDM5D	16.1	ND	ND
	NLGN4Y	−5.9	−5.8	NS
	TGIF2LY	−8.9	ND	ND
	TSPY3	−6.9	ND	ND
	VCY	10.3	ND	NS
	XKRY2	−6.3	ND	ND
	ZFY	−6.6	NS	NS
FOE vs IOE	AMELY	−15.2	ND	ND
	BPY2B	−8.0	ND	ND
	CDY2A	−9.3	ND	ND
	EIF1AY	2.1	2.5	2.4
	HSFY2	−21.8	ND	ND
	NLGN4Y	4.6	4.3	NS
	PRKY	13.9	NS	NS
	TBL1Y	2.8	ND	ND
	TGIF2LY	−73.9	ND	ND
	VCY	−7.5	ND	NS
	ZFY	−2.3	NS	NS
FCON vs FOE	AMELY	−4.8	ND	ND
	KDM5D	10.0	ND	ND
ICON vs IOE	AMELY	−11.2	ND	ND
	BAGE	9.8	NS	3.5
	DDX3Y	−11.3	ND	ND
	DAZ2	−2.4	ND	ND
	EIF1AY	2.1	5.1	2.9
	KDM5D	−25.6	ND	ND
	NLGN4Y	2.2	3.7	NS
	PRKY	11.4	NS	NS
	TGIF2LY	−9.3	ND	ND
	VCY	−38.3	ND	NS

FCON, control, fertile; ICON, control, infertile; FOE, stage IV, ovarian endometriosis, fertile; IOE, stage IV, ovarian endometriosis, infertile. ND, not detected. NS, not significant. AMELY, amelogenin Y-linked; BPY2B, basic charge Y-linked 2B; BAGE, B melanoma antigen; CD24, small cell lung carcinoma cluster 4 antigen; CDY2A, chromodomain Y-linked 2A; DAZ2, deleted in azoospermia 2; DDX3Y, DEAD-box helicase 3 Y-linked; EIF1AY, eukaryotic translation initiation factor 1A Y-linked; HSFY2, heat shock transcription factor Y-linked 2; KDM5D, lysine demethylase 5D; NLGN4Y, neuroligin 4 Y-linked; PRKY, protein kinase Y-linked; TBL1Y, transducin beta-like 1 Y-linked; TGIF2LY, TGFB induced factor homeobox 2-like Y-linked; TSPY3, testis specific protein Y-linked 3; VCY, variable charge Y-linked; XKRY2, X-Kell blood group precursor-related Y-linked 2; ZFY, zinc finger protein Y-linked

### Target DNA inserts

Quantitative PCR for identifying DNA targets for nine (9) MSY-linked genes revealed positive results for seven (7) genes in various samples, two (KDM5D and SRY) however were undetectable (Table 4). Furthermore, the prevalence and concentration of PCR-positive target inserts displayed generally higher trends in the fertile, control (FCON) patients compared with the other three groups (ICON, FOE and IOE); however, significantly higher values were obtained in the fertile, control (FCON) patients only when compared with the fertile, OE patients (FOE) for the prevalence of BAGE, PRKY, and TTTY9A and for the concentration of ZFY.

**Table 4:** Profile of Y chromosome-linked specific gene-associated DNA inserts in endometrial samples

Target	Total number of samples with positive insert/Total number of samples (Range of copy numbers)1			
	Control (CON)		Endometriosis OE)	
	Fertile (FCON)	Infertile (ICON)	Fertile (FOE)	Infertile (IOE)
BAGE	6/6	5/9	0/9**	9/9
	(3.5–3.7)	(2.4–3.2)		(2.0–3.1)
EIF1AY	6/6	5/9	4/9	6/9
	(2.1–3.5)	(2.4–3.3)	(3.0–3.4)	(1.1–3.1)
KDM5D^2^	0/6	0/9	0/9	0/9
NLGN4Y	6/6	6/9	6/9	6/9
	(2.4–3.3)	(2.4–3.9)	(3.3–3.6)	(1.2–3.0)
PRKY	6/6	3/9	0/9**	2/9
	(2.1–2.5)	(1.4–2.1)		(0.1–1.6)
SRY	0/6	0/9	0/9	0/9
TTTY9A	6/6	3/9	0/9**	2/9
	(0.8–0.9)	(0.8–1.0)		(−0.6–0.4)**
TTTY14	6/6	9/9	9/9	9/9
	(3.6–3.7)	(3.6–3.7)	(3.5–3.7)	(3.5–3.7)
ZFY	6/6	9/9	3/9	9/9
	(3.2–3.5)	(2.9–3.5)	(1.0–2.7)*	(3.2–3.4)

^1^In logarithmic scale. No detectable amplifications and very high Cq values (> 35) were considered as not detectable and shown as 0-value. ^2^Variants 1–3. *P < 0.05, **P < 0.02 compared with group 1A. BAGE, B melanoma antigen; KDM5D, lysine demethylase 5D; PRKY, protein kinase Y-linked; SRY, sex determining region Y; TTTY9A, testis-specific transcript Y-linked 9A; TTTY14, testis-specific transcript Y-linked 14; ZFY, zinc finger protein Y-linked. The prevalence data were tested using Fisher’s exact test and the copy number data were tested using Kruskal-Wallis followed by the Mann-Whitney U-test

## Discussion

A panel of molecular biology tools including quantitative PCR, microarray expression, RNA-seq, and real time RT-PCR were employed to examine the Y chromosome microchimerism in the endometrium using secretory phase samples from fertile and infertile patients with severe OE (stage IV) and without endometriosis. This experimental strategy was adopted since earlier studies using FISH yielded conflicting results [14, 15]. FISH is a relatively less sensitive technique and it probes into a very small portion of the sample [22–25]. Furthermore, two probes used in the previous FISH study to detect the Y chromosome in endometrial cells were directed against the centromeric (Yp11.1 to Yq11.1) and telomeric (Yq12 satellite) regions of the Y chromosome [15]. Both these regions are gene-sparse heterochromatised regions of the Y chromosome. In the present study, the DNA segments detected are from the ampliconic regions of the Y chromosome. Moreover, it is generally believed that chromosome-centric quantitative expression data bear robust relevance towards understanding the deep physiology of cells and tissues [26, 27].

A consolidated summary of the results of the present study is presented in Table 5. The results of our study revealed that male microchimerism in the endometrium is widely present in all groups of patients irrespective of endometriosis and pregnancy history. A large number of reports indeed substantiate the notion of the widespread presence of male microchimerism in human females [28–32].

**Table 5:** Summary of observations from quantitative PCR (qPCR), expression microarray (MA), RNA-seq (RS) and qRT-PCR (RT) for endometrial Y chromosome microchimerism

Gene name	qPCR				MA				RS/RT			
	CON		OE		CON		OE		CON		OE	
	F	I	F	I	F	I	F	I	F	I	F	I
BAGE	+	+/−	_	+	3	3	3	1	+	+	+	+
EIF1AY	+	+/−	−/+	+/−	2	3	3	2	+	+	+	+
NLGN4Y	+	+/−	+/−	+/−	3	3	3	3	+	+	+	+
PRKY	+	−/+	_	−/+	2	3	3	2	+	+	+	+
TTTY9A	+	−/+	_	−/+	2	3	1	4	_	_	_	_
TTTY14	+	+	+/−	+	2	3	2	4	_	_	_	_
ZFY	+	+	−/+	+	3	3	3	3	+	+	+	+

F, fertile. I, infertile. OE, ovarian endometriosis. CON, control endometrium. +, detected in ≥76% samples; +/−, detected in ≥50% but ≤75% samples; −/+, detected in < 50% samples; _, not detected in any sample. 1, very low; 2, low; 3, moderate; 4, high; 5, very high

It is apparent from the present study that Y chromosome microchimerism in the endometrium in general did not show any marked relationship with pregnancy history, as it was observed in both fertile and infertile patients with and without disease. Furthermore, the assumption that Y chromosome microchimerism evolves with a higher prevalence in women from pregnancies with sons [33–35] could not be empirically assessed in the present study as half of the fertile women had at least one son and one-fifth declined to report the details of the gender of their offspring. However, we did not observe any marked indication of such association from the present set of data. Indeed, earlier reports indicated that male microchimerism was seen among women without any son; in fact, 10% of them had never been pregnant before [36]. It has been suggested that microchimerism is not only limited to the bi-directional exchange of cells between mother and foetus but also cells from older siblings and even maternal grandmother cells may also be transferred to the foetus and can persist in various tissues for a long period of time [22, 33, 37–40].

Furthermore, our study reveals interesting observations regarding the prevalence and concentration of observed DNA inserts of the Y chromosome, particularly *vis-à-vis* their transcriptional expression in the endometrium. PCR experiments for quantifying DNA inserts of the Y chromosome revealed a lower prevalence and concentration of male microchimerism in diseased eutopic tissues; however, their steady state expressions were higher. In contrast, the prevalence and concentration of male microchimerism was higher with lower transcript expressions in the control endometrial samples. Chan et al. also observed a lower prevalence and concentration of male microchimerism in the brains of women with Alzheimer’s disease when compared to the brains of women without neurologic disease [34]. Sawaya et al. observed, using quantitative PCR, that all systemic sclerosis (SSc) samples were positive for male DNA compared with higher levels of microchimerism in clinically unaffected SSc skin [22]. In fact, several groups have indicated the possibility that foetal microchimerism may actually render a benefit to the mother’s health in certain conditions [32, 39, 41–46].

**Fig. 3:**
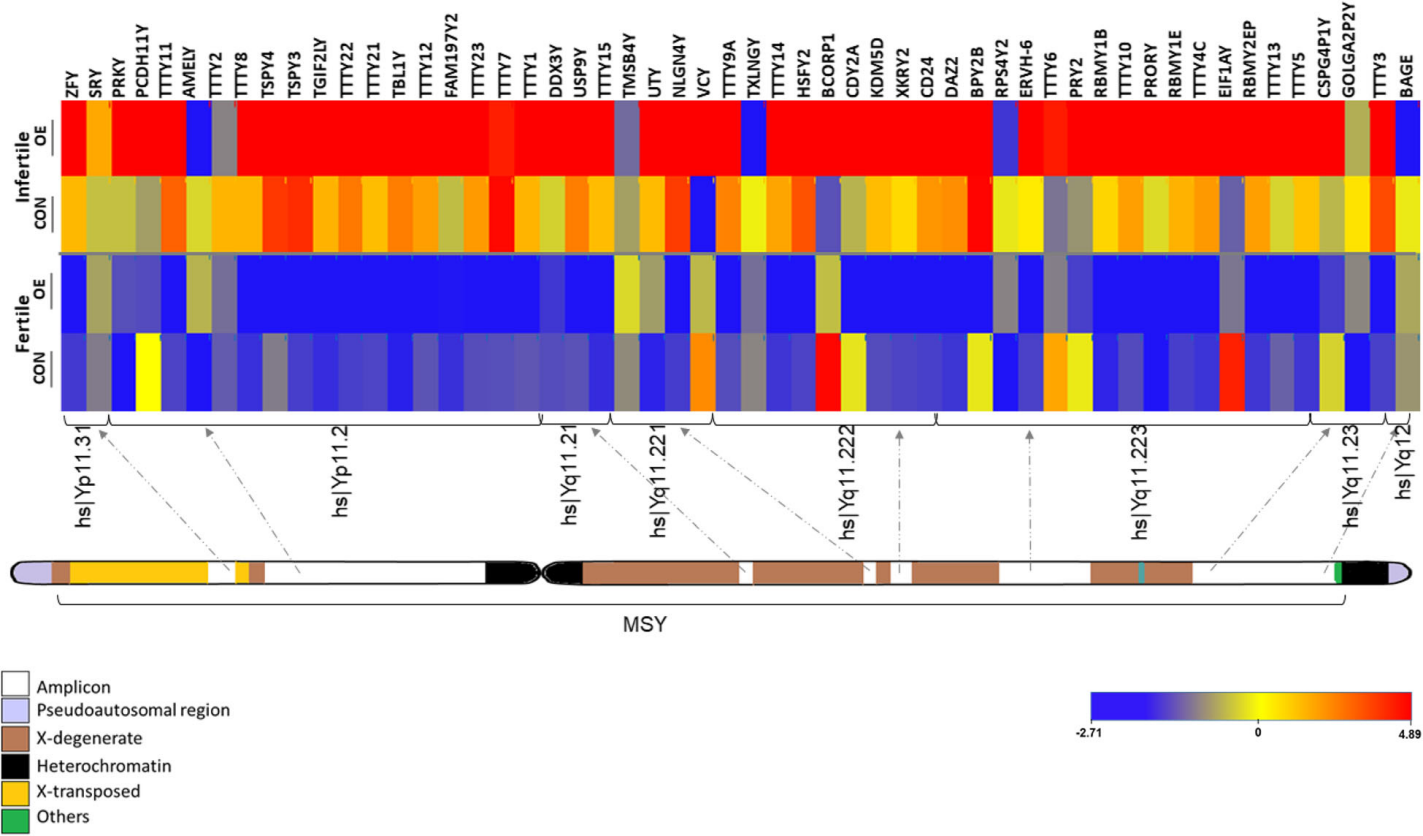
Physical map of human male-specific region of the Y chromosome (MSY) with markings of cytogenic bands for genes in ampliconic sequences that were observed to display very high to very low levels of transcript expression (as shown by colour codes) in expression microarray experiments with endometrial samples obtained from fertile and infertile women of control (CON) and ovarian endometriosis (OE) groups. Heat map depicts mean transcript expressions of MSY-linked genes detected in whole genome expression microarrays with secretory phase endometrium. Different colour codes in boxes for different classes of sequences in MSY-linked genes, and colour spectrum for expression ranges are shown

Figure 3 provides the physical map of the 54 genes detected by microarray in a typical Y chromosome. According to the available database, the Y chromosome is approximately 60 Mb in size and consists of the male-specific region of the Y chromosome (MSY), which contains 73 protein-coding genes and 122 non-coding RNA genes and two small pseudoautosomal regions flanking each side (https://www.ncbi.nlm.nih.gov). Based on gene ontology (GO) analysis, the molecular function of proteins potentially arising from those 54 microarray-positive transcripts of the Y chromosome in patients can be predicted. Those predications are categorised into protein binding, DNA binding, RNA binding, metal ion binding, transcription factor activity, transcription regulator activity, transferase activity, ATP binding, translation activity, esterase activity, oxidoreductase activity, kinase activity, protease activity and helicase activity (https://www.ebi.ac.uk/GOA). In terms of biological processes in GO analysis, the proteins of those transcripts are involved in transcription, cell differentiation, gonad development, metabolic processes, tissue development, nucleosome assembly, chromatin modification, translation, sex differentiation, cell adhesion, RNA metabolism, cell proliferation and sex determination (https://www.ebi.ac.uk/GOA).

In the present study, a few Y chromosome-linked long noncoding RNAs (lncRNAs) in the endometrium were detected at both the DNA and RNA levels. As expected, their signals were generally in the lower scale, except for TTTY14 which displayed a high order of prevalence and concentration in all samples. The significance of this observation is open to further study. It is now well acknowledged that the human genome contains a large number of lncRNAs and that this subset of non-coding RNAs accomplish a remarkable variety of biological functions [47–50]. Although there is a growing interest about the potential role of various lncRNAs in endometriosis and infertility, [51–53] and more specifically the possible association between Y chromosome-linked lncRNAs (for example, lncKDM5D) and fat metabolism and cellular inflammation in the causation of atherosclerosis and CAD in men, [46] we have no understanding about the possible involvement of specifically the MSY-linked lncRNAs in endometriosis and in infertility.

It appears intriguing that several coding (AMELY, PCDH11, SRY, TGIF2LY, TSPY3, and USP9Y) and non-coding (TTTY2, TTTY4C, TTTY5, TTTYY6, TTTY8, TTTY10, TTTY14, TTTY21, TTTY22, and TTTY23) genes commonly displayed low expression in samples from fertile patients and high expression in samples from infertile patients with very little influence of OE on their expression profile. It appears that MSY-linked transcript expressions showed marked association with infertility. In this regard, it is noteworthy that mere microchimerism may be of minimal consequence, as it is rather a frequent phenomenon in women who have carried foetuses, as well as, in nulliparous women via maternal origin. Additional combinatorial events in the form of bacterial, viral, chemical or other types of challenge are suggestively necessary to activate microchimeric cells to result in lesions [22, 32]. Additionally, the observation that higher male microchimerism at the level of the MSY-linked ampliconic sequence, however, with lower ranges of transcript expressions in control, fertile patients compared to patients in other groups appears interesting.

Despite an apparent empirical confirmation in the form of qPCR and expression microarray data, the quantitative data obtained from RNA and DNA methods failed to show marked mutual congruencies in the present study, the basis of which was not explored. However, such lack of concordance has earlier been reported in other systems [54]. Moreover, such incongruences may arise from a lack of linear quantitative correspondence in the central flow from DNA to RNA in steady states, which may potentially play an important adjunct pathophysiological role in various diseases and conditions [55, 56]. The steady state data recorded in the present study can neither investigate this complex issue nor reveal the hierarchy of cause and effect relationship between male microchimerism and endometriosis or infertility. Further functional studies are required in order to gather large scale dynamic sets of data yielding knowledge in this regard, which may be of significance for understanding endometriosis and infertility.

## Conclusions

In conclusion, this is the first report with a general observation that several coding and non-coding genes of MSY origin displayed microchimerism in the form of presence of their respective DNA inserts along with their microarray-detectable expression. This expression was especially high in infertile women, and even higher in infertile patients with OE compared with fertile patients with and without endometriosis. Further chromosome-centric human proteomics and other functional studies are required to delineate how various routes of microchimerism indeed work in the pathophysiology of endometriosis and/or infertility.

## Additional files

Additional file 1: **Table S1.** List of genes and their primers used for quantitative PCR and qRT-PCR. (DOC 33 kb)

Additional file 2: **Table S2.** List of genes with their microarray expression levels from very low (− 3.5) to very high (5.0) in the different groups. (DOCX 43 kb)
