# Supplementary material for: Genomic evidence of Y chromosome microchimerism in the endometrium during endometriosis and in cases of infertility
Source: Reprod Biol Endocrinol. 2019 Feb 13;17:22. doi: 10.1186/s12958-019-0465-z (PMC6375207; doi:10.1186/s12958-019-0465-z)
Supplement: Supplementary file 1 — Table S1. List of genes and their primers used for quantitative PCR and qRT-PCR. (DOC 33 kb) [file 12958_2019_465_MOESM1_ESM.doc]

Table S1. List of genes and their primers used for quantitative PCR

______________________________________________________________________________

Gene name Accession number Primer sequences^1^

(*Gene symbol*) (RefSeq)

_______________________________________________________________________________

Amelogenin, Y-linked NM_001143 ACCTCTGCCTCCAATGTTC (s)

(*AMELY*) ATCCACTTCCTCCTGCTTG (as)

B melanoma antigen AF527552 CAGGAAGAAGAGGAGGAG (s)

(*BAGE*) TGTAGCAGATAGTCATTGTC (as)

Basic charge, Y-linked, 2B NM_001002760 CCTGGATGATGTTACTCTTC (s)

(*BPY2B*) AATATAATCTTGGTGATGTGAC (as)

Beta actin NM_001033084 GTGCGTGACATTAAGGAG (s)

(*ACTB*)^2^ AGGAAGGAAGGTTGGAAG (as)

Beta globin NP_000509.1 TCTGTCCACTCCTGATGCTG (s)

*(HBB)*^2^ GAAAACATCAAGCGTCCCAT (as)

Beta-2-microglobulin NM_001047137 AGATGTCTCGCTCAGTGG (s)

(*B2M*)^2^ AGTCAACTTCAATATCAGATGG (as)

CD24 molecule XM_002799072 TGCTGGCACTGCTCCTAC (s)

(*CD24*) CGAAGAGACTGGCTGTTGAC (as)

Chromodomain protein, NM_001198733 CACTCTACCTCATAATCATACAG (s)

Y-linked, 2A (*CDY2A*) TTCATTCGCCTTCTTAATACC (as)

Glyceraldehyde 3 phosphate NM_002046 CGACAACGAATATGGCTA (s)

dehydrogenase (*GAPDH*)^2^ CTGGGAAGGAAAGAAGGG (as)

Deleted in azoospermia 2 XM_001094309 GTTAATCCTCCTCCTCCAC (s)

(*DAZ2*) ATATACAAGCAACTGACATCC (as)

DEAD (Asp-Glu-Ala-Asp) AF000984 TGTAGGTCCAGTGTAAGAGTTC (s)

box helicase 3, Y-linked AGAGTTCAGGTCCAGATTAGC (as)

(*DDX3Y)*

Eukaryotic translation NM_004681 CTTAGTTCAGTCGGCTCTTAG (s)

initiation factor 1A, GCATACTCTTGTCCATCCTC (as)

Y-linked (*EIF1AY*)

Heat shock transcription NM_153716 TTGGGCTGACAGTGGAAC (s)

factor, Y linked 2 CAGTGGTGATGGTTGAAGAG (as)

transferase (*HSFY2*)

Lysine (K)-specific NM_004653 TGCGAATCAGAAGACACAGC (s)

demethylase 5D (*KDM5D*) TGGAACACAGACGATAAGAACG (as)

Phophatidyl inositol JV043498 GGAACAGAAGGAGAAGGAG (s)

glycan anchor biosynthesis GATAGAAGTCGGAGAAGAGG (as)

receptor V (*NLGN4Y*)

Protein kinase, Y-linked, NR_028062 CTTCTCACTCACCATCCTGTTG (s)

pseudogene (*PRKY*) GCTAATCTCCTCTGTGTCATTCC (as)

Proline rich, Y-linked NM_001282471 GTGATGATGAGGAGGAGTC (s)

beta receptor II (*PRORY*) GAGAGCAATGAGGACAGG (as)

Sex determining region Y NM_003140 CAACAGCGATGATTACAGTC (s)

(*SRY*) AGACCACACGATGAATGC (as)

TGFB-induced factor NM_139214 CTCTCCTGAACACTGATG (s)

homeobox 2-like, Y-linked TGATGATGATGCTCTTGG (as)

(*TGIF2LY*)

Testis Specific Protein, NM_001077697 CTGGAGGTGGAAGAAGAGAAGC (s)

Y-Linked 3 GGTGTCTGCGGCGATACG (as)

*(TSPY3)*

Testis-specific transcript NR_001530 GACCTATTAGTGCCTTGA (s)

Y-linked 9A (TTTY9A) AGTATGTTCCTGATGCTATA (as)

Testis-specific transcript NR_001543 GGCTGAGGCTGAAGGAATA (s)

Y-linked 14 (TTTY14) TTAAAGTCAGGGTCTCACTATGT (as)

Ubiquitously transcribed NM_007125 ACGGTGGTCTTCAAGGTG (s)

tetratricopeptide repeat GACATCTGCGACATTAGTGG (as)

containing, Y-linked *(UTY)*

Variable charge, Y-linked NM_004679 GCAGCCTGGAGTTAGTCG (s)

*(VCY)*  CCTTGGTAGTCTTCTTCTTCG (as)

XK related, Y-linked NM_001002906 ATGGTGGGTAAGTATGGATG (s)

*(XKRY2)*  CTGTAGTGTAGGATTGTATGTC (as)

Zinc finger protein, NM_003411 GCTCTGTGCTTCATCTTCTG (s)

Y-linked *(ZFY)* ACGGTTACCCTCCTATTGC (as)

___________________________________________________________________________

^1^primers were designed using Beacon Designer Software (Premier Biosoft, Palo Alto, CA, USA) based on gene sequences corresponding to the genes retrieved from NCBI data base. ^2^housekeeping genes. as, anti-sense. s, sense.
