# Supplementary material for: Genomic evidence of Y chromosome microchimerism in the endometrium during endometriosis and in cases of infertility
Source: Reprod Biol Endocrinol. 2019 Feb 13;17:22. doi: 10.1186/s12958-019-0465-z (PMC6375207; doi:10.1186/s12958-019-0465-z)
Supplement: Supplementary file 2 — Table S2. List of genes with their microarray expression levels from very low (− 3.5) to very high (5.0) in the different groups. (DOCX 43 kb) [file 12958_2019_465_MOESM2_ESM.docx]

Table S2. List of genes with their microarray expression levels from very low (-3.5) to very high (5.0) in the different groups*

___________________________________________________________________________

**FCON (Control, fertile)**

Low ranges (<-2.0 to -0.5)

| Serial No. | Gene  symbol | Normalized expression |
| --- | --- | --- |
|  | *GOLGA2P2Y* | -1.75443 |
|  | AMELY | -1.67183 |
|  | *PRKY* | -1.46798 |
|  | RBMY2EP | -1.2729 |
|  | EIF1AY | -1.21545 |
|  | *TTTY2* | -1.11024 |
|  | TMSB4Y | -1.0972 |
|  | RBMY1B | -1.07247 |
|  | *TTTY14* | -1.01514 |
|  | USP9Y | -1.00628 |
|  | *TTTY1* | -1.00134 |
|  | TGIF2LY | -0.97685 |
|  | *TTTY22* | -0.97564 |
|  | *TTTY10* | -0.94415 |
|  | BPY2B | -0.93445 |
|  | CDY2A | -0.93223 |
|  | *TTTY6* | -0.92786 |
|  | *TTTY4C* | -0.92709 |
|  | SRY | -0.89651 |
|  | UTY | -0.87122 |
|  | RPS4Y2 | -0.85844 |
|  | TSPY4 | -0.85388 |
|  | *TTTY11* | -0.85055 |
|  | XKRY2 | -0.85014 |
|  | *TTTY3* | -0.83861 |
|  | ERVH-6 | -0.83214 |
|  | PCDH11Y | -0.81597 |
|  | HSFY2 | -0.81445 |
|  | *TTTY12* | -0.80872 |
|  | TXLNGY | -0.80819 |
|  | RBMY1E | -0.75216 |
|  | *TTTY9A* | -0.73833 |
|  | DDX3Y | -0.73507 |
|  | *BCORP1* | -0.7341 |
|  | *TTTY23* | -0.72725 |
|  | *TTTY7* | -0.71651 |
|  | *TTTY15* | -0.68724 |
|  | *TTTY21* | -0.67211 |
|  | *TTTY8* | -0.65615 |
|  | TBL1Y | -0.61727 |
|  | *TTTY5* | -0.56103 |

Moderate ranges (<-0.5 to 2.0)

| Serial No. | Gene  symbol | Normalized expression |
| --- | --- | --- |
|  | ZFY | -0.34227 |
|  | TSPY3 | -0.33477 |
|  | CD24 | -0.26727 |
|  | NLGN4Y | -0.21557 |
|  | BAGE | -0.12414 |
|  | CSPG4P1Y | 0.393464 |
|  | DAZ2 | 0.564255 |
|  | PRY2 | 0.590603 |
|  | FAM197Y2 | 0.764326 |
|  | PRORY | 1.262876 |
|  | VCY | 1.414479 |

High ranges (>2.0 to 3.5)

| Serial No. | Gene  symbol | Normalized  expression |
| --- | --- | --- |
|  | *TTTY13* | 2.011258 |
|  | KDM5D | 2.146298 |

**ICON (Control, infertile)**

Low ranges (<-2.0 to -0.5)

| Serial No. | Gene  symbol | Normalized  expression |
| --- | --- | --- |
|  | VCY | -2.00693 |
|  | KDM5D | -0.68667 |
|  | *TTTY13* | -0.56424 |

Moderate ranges (<-0.5 to 2.0)

| Serial No. | Gene  symbol | Normalized  expression |
| --- | --- | --- |
|  | PRORY | -0.40216 |
|  | PRY2 | -0.13774 |
|  | FAM197Y2 | -0.04931 |
|  | NLGN4Y | -0.03302 |
|  | CSPG4P1Y | 0.159031 |
|  | *TTTY22* | 0.215949 |
|  | AMELY | 0.240978 |
|  | ZFY | 0.243845 |
|  | DDX3Y | 0.383296 |
|  | *TTTY5* | 0.402583 |
|  | *PRKY* | 0.428915 |
|  | RBMY2EP | 0.465947 |
|  | EIF1AY | 0.563752 |
|  | BAGE | 0.592345 |
|  | CD24 | 0.640495 |
|  | ERVH-6 | 0.88044 |
|  | *GOLGA2P2Y* | 0.924923 |
|  | TXLNGY | 0.980264 |
|  | RBMY1B | 1.000585 |
|  | *TTTY6* | 1.101369 |
|  | *TTTY12* | 1.134565 |
|  | *TTTY9A* | 1.139126 |
|  | USP9Y | 1.143103 |
|  | TMSB4Y | 1.146963 |
|  | SRY | 1.16436 |
|  | TBL1Y | 1.180379 |
|  | *TTTY8* | 1.184949 |
|  | TGIF2LY | 1.186112 |
|  | *TTTY1* | 1.189311 |
|  | RPS4Y2 | 1.190937 |
|  | *TTTY14* | 1.235211 |
|  | CDY2A | 1.268057 |
|  | RBMY1E | 1.293816 |
|  | *TTTY4C* | 1.297412 |
|  | *TTTY21* | 1.30589 |
|  | *TTTY23* | 1.327662 |
|  | *TTTY10* | 1.337751 |
|  | XKRY2 | 1.352962 |
|  | BPY2B | 1.395424 |
|  | *BCORP1* | 1.460563 |
|  | *TTTY2* | 1.486796 |
|  | *TTTY15* | 1.487283 |
|  | *TTTY11* | 1.510962 |
|  | PCDH11Y | 1.63947 |
|  | HSFY2 | 1.733505 |
|  | *TTTY3* | 1.768053 |
|  | UTY | 1.851238 |
|  | TSPY3 | 1.878539 |
|  | TSPY4 | 1.943255 |

High ranges (>2.0 to <3.5)

| Serial No. | Gene  symbol | Normalized expression |
| --- | --- | --- |
|  | *TTTY7* | 2.143113 |
|  | DAZ2 | 2.167818 |

**FOE (Endometriosis, fertile)**

Very Low ranges (-3.5 to -2.0)

| Serial No. | Gene  symbol | Normalized  expression |
| --- | --- | --- |
|  | TXLNGY | -2.5116 |
|  | RBMY1B | -2.43944 |
|  | XKRY2 | -2.31172 |
|  | *TTTY7* | -2.27024 |
|  | *TTTY9A* | -2.26442 |
|  | TSPY4 | -2.24562 |
|  | *TTTY12* | -2.22403 |
|  | *TTTY23* | -2.21426 |
|  | *TTTY11* | -2.19596 |
|  | CDY2A | -2.15237 |
|  | *BCORP1* | -2.12173 |
|  | UTY | -2.11314 |
|  | *TTTY1* | -2.08525 |
|  | *TTTY15* | -2.07386 |
|  | SRY | -2.07111 |

Low ranges (<-2.0 to -0.5)

| Serial No. | Gene  symbol | Normalized  expression |
| --- | --- | --- |
|  | DAZ2 | -1.99848 |
|  | RBMY2EP | -1.9953 |
|  | *TTTY4C* | -1.98283 |
|  | ERVH-6 | -1.96664 |
|  | USP9Y | -1.95836 |
|  | RBMY1E | -1.93889 |
|  | *TTTY14* | -1.93231 |
|  | BPY2B | -1.93187 |
|  | TGIF2LY | -1.9215 |
|  | HSFY2 | -1.88581 |
|  | RPS4Y2 | -1.86046 |
|  | *TTTY8* | -1.85838 |
|  | *TTTY21* | -1.84748 |
|  | *TTTY6* | -1.8375 |
|  | TSPY3 | -1.79847 |
|  | *TTTY5* | -1.59689 |
|  | *TTTY10* | -1.51988 |
|  | *TTTY2* | -1.48632 |
|  | *TTTY3* | -1.36908 |
|  | PCDH11Y | -1.26209 |
|  | *TTTY22* | -1.1896 |
|  | DDX3Y | -0.91798 |
|  | CSPG4P1Y | -0.86491 |
|  | PRY2 | -0.85001 |
|  | FAM197Y2 | -0.74596 |
|  | AMELY | -0.71614 |

Moderate ranges (<-0.5 to 2.0)

| Serial No. | Gene  symbol | Normalized  expression |
| --- | --- | --- |
|  | CD24 | -0.45656 |
|  | TBL1Y | -0.45217 |
|  | *TTTY13* | -0.38702 |
|  | PRORY | -0.32193 |
|  | EIF1AY | -0.28445 |
|  | *GOLGA2P2Y* | -0.25012 |
|  | TMSB4Y | -0.06081 |
|  | ZFY | -0.01523 |
|  | BAGE | 0.023103 |
|  | *PRKY* | 0.12737 |
|  | VCY | 0.174985 |
|  | KDM5D | 0.217889 |
|  | NLGN4Y | 0.442487 |

**IOE (Endometriosis, infertile)**

Very low ranges (-3.5 to -2.0)

| Serial No. | Gene  symbol | Normalized expression |
| --- | --- | --- |
|  | CD24 | -2.71243 |
|  | BAGE | -2.71182 |

Low ranges (<-2.0 to -0.5)

| Serial No | Gene  symbol | Normalized expression |
| --- | --- | --- |
|  | *PRKY* | -1.95548 |
|  | EIF1AY | -0.92281 |

Moderate ranges (<-0.5 to 2.0)

| Serial No | Gene  symbol | Normalized expression |
| --- | --- | --- |
|  | NLGN4Y | -0.48163 |
|  | TBL1Y | -0.28238 |
|  | *GOLGA2P2Y* | 0.083387 |
|  | ZFY | 1.252479 |

High ranges (>2.0 to 3.5)

| Serial No | Gene  symbol | Normalized expression |
| --- | --- | --- |
|  | *TTTY7* | 2.016883 |
|  | PRORY | 2.037395 |
|  | TXLNGY | 2.185001 |
|  | TMSB4Y | 2.232742 |
|  | RPS4Y2 | 2.303156 |
|  | DDX3Y | 2.30863 |
|  | DAZ2 | 2.338005 |
|  | *TTTY1* | 2.349504 |
|  | XKRY2 | 2.360194 |
|  | RBMY1E | 2.431302 |
|  | HSFY2 | 2.431856 |
|  | VCY | 2.444441 |
|  | *TTTY15* | 2.457066 |
|  | *TTTY23* | 2.492229 |
|  | TSPY4 | 2.522977 |
|  | *TTTY9A* | 2.535972 |
|  | UTY | 2.578688 |
|  | BPY2B | 2.607154 |
|  | USP9Y | 2.644974 |
|  | *TTTY21* | 2.669407 |
|  | *TTTY3* | 2.755117 |
|  | *TTTY10* | 2.766012 |
|  | *TTTY12* | 2.830734 |
|  | ERVH-6 | 2.836303 |
|  | *TTTY5* | 2.893486 |
|  | CDY2A | 2.963795 |
|  | *BCORP1* | 3.016231 |
|  | *TTTY22* | 3.021953 |
|  | *TTTY4C* | 3.039553 |
|  | *TTTY11* | 3.055061 |
|  | *TTTY14* | 3.092497 |
|  | PCDH11Y | 3.195688 |
|  | *TTTY6* | 3.302937 |
|  | AMELY | 3.329095 |

Very high ranges (>3.5 to 5.0)

| Serial No | Gene  Symbol | Normalized expression |
| --- | --- | --- |
|  | SRY | 3.548874 |
|  | *TTTY8* | 3.567476 |
|  | CSPG4P1Y | 3.578502 |
|  | *TTTY13* | 3.60142 |
|  | *TTTY2* | 3.621706 |
|  | PRY2 | 3.626952 |
|  | FAM197Y2 | 3.77163 |
|  | RBMY1B | 3.791823 |
|  | KDM5D | 3.931224 |
|  | TSPY3 | 4.006855 |
|  | TGIF2LY | 4.598791 |
|  | RBMY2EP | 4.883164 |

___________________________________________________________________________

Non-coding genes are shown in *italics*. *Data archived at GSE120103.
